# Versatile Role of Prokineticins and Prokineticin Receptors in Neuroinflammation

**DOI:** 10.3390/biomedicines9111648

**Published:** 2021-11-09

**Authors:** Roberta Lattanzi, Rossella Miele

**Affiliations:** 1 Department of Physiology and Pharmacology “Vittorio Erspamer”, Sapienza University of Rome, Piazzale Aldo Moro 5, I-00185 Rome, Italy; 2 Department of Biochemical Sciences “A. Rossi Fanelli”, CNR Institute of Molecular Biology and Pathology, Sapienza University of Rome, Piazzale Aldo Moro 5, I-00185 Rome, Italy

**Keywords:** neuroinflammation, prokineticins, GPCR, blood–brain barrier

## Abstract

Prokineticins are a new class of chemokine-like peptides involved in a wide range of biological and pathological activities. In particular, prokineticin 2 (PK2), prokineticin receptor 1 (PKR1) and prokineticin receptor 2 (PKR2) play a central role in modulating neuroinflammatory processes. PK2 and PKRs, which are physiologically expressed at very low levels, are strongly upregulated during inflammation and regulate neuronal-glial interaction. PKR2 is mainly overexpressed in neurons, whereas PKR1 and PK2 are mainly overexpressed in astrocytes. Once PK2 is released in inflamed tissue, it is involved in both innate and adaptive responses: it triggers macrophage recruitment, production of pro-inflammatory cytokines, and reduction of anti-inflammatory cytokines. Moreover, it modulates the function of T cells through the activation of PKR1 and directs them towards a pro-inflammatory Th1 phenotype. Since the prokineticin system appears to be upregulated following a series of pathological insults leading to neuroinflammation, we will focus here on the involvement of PK2 and PKRs in those pathologies that have a strong underlying inflammatory component, such as: inflammatory and neuropathic pain, Alzheimer’s disease, Parkinson’s disease, multiple sclerosis, stroke, obesity, diabetes, and gastrointestinal inflammation.

## 1. Introduction

The central nervous system (CNS) has always been considered a district protected from inflammatory processes due to the presence of the blood–brain barrier (BBB). It was thought that inflammation could only be caused by the rupture of this with the consequent infiltration of immune cells. It has recently been shown that CNS inflammation is a much more complex process called “neuroinflammation”. This term indicates the activation of microglia, astrocytes, neurons and endothelial cells that induce an alteration of the permeability of the BBB. This results in increased leukocyte infiltration in the brain, increased secretion of proinflammatory cytokines and chemokines, and ultimately, neuronal damage and death [1]. Neuroinflammation plays a central role in the development and progression of many neurodegenerative diseases such as Parkinson’s disease, psychiatric and behavioral disorders, multiple sclerosis, and Alzheimer’s disease.

During neuroinflammation, to the three main occurring processes represented by the weakening of synapses, the inhibition of neurogenesis and the death of neurons actively participate those who are now considered the main actors of neuroinflammation: neurons, microglia and astrocytes. Indeed, it has been definitively established that neurons, traditionally thought to be passive bystanders in neuroinflammation, are capable of producing various mediators, such as prostanoids, cytokines (such as IL-1β, IL-6 and TNF-α) and inducible enzymes (iNOS) [2]. Also microglia are a central player in neuroinflammation and interact with other cell types that regulate neurodegeneration. Depending on the acquired phenotype, microglial cells may play a positive or negative role in neuronal survival. Inflammatory microglia exhibit the M1 phenotype and stimulate astrocyte activation, neuronal damage, T cell activation, and BBB disruption, all effects that promote neuroinflammation and subsequent neuronal loss. Conversely, microglia exhibiting an M2-type anti-inflammatory phenotype attenuate the inflammation and neurotoxic effects induced by M1-type microglia by supporting neuron survival, limiting barrier damage, and promoting repair of damaged tissue [3].

Finally, under homeostatic conditions, astrocytes have been demonstrated to play a role in several crucial biological processes. They support BBB endothelial cells, help maintain ionic balance and pH, contribute to synaptogenesis, modulate information processing and signal transduction, regulate synaptic and neuronal plasticity, deliver nutrients to neuro-glial cells, maintain excitability and neuronal network connectivity [4].

Conversely, during neuroinflammation, astrocytes become activated following stimulation by pro-inflammatory mediators, which, depending on the phenotype acquired, can affect any of these highly regulated processes. In LPS-induced neuroinflammation astrocytes, acquire an A1 neurotoxic phenotype loosing normal function, such as the ability to induce synapse formation. Other insults, such as ischemia, can induce a protective A2 reactive astrocyte to secrete neuroprotective molecules that promote CNS recovery and repair. Importantly, recent transcriptome analysis studies have demonstrated that reactive astrocytes can exhibit a gradient of phenotypes akin to activated microglia and T lymphocytes. As such, it is not possible to make a clear distinction between the two previously established activation states (A1 vs. A2) [5].

Prokineticins belong to a family of small secretory proteins that are highly conserved in evolution from invertebrates to humans. The first member of this family to be isolated was a nontoxic component of black mamba snake venom called Mamba Intestinal Toxin 1 (MIT-1) because it caused contraction of the guinea pig ileum [6]. In the same years, a small protein of 77 amino acids was isolated from the skin secretions of the frog *Bombina variegata* and named Bv8 [6]. Later, two human Bv8-like proteins were identified and named prokineticin 1 (PK1, or EG-VEGF) and prokineticin 2 (PK2, or mammalian Bv8) based on their ability to trigger contraction of the guinea pig ileum [6]. These proteins share some structural features: an amino-terminal AVITGA peptide segment important for biological activity and receptor recognition, ten cysteine residues forming five disulfide bridges, and a tryptophan residue at position 24 essential for binding to prokineticin receptors [7,8]. These are two related G protein-coupled receptors (PKR1 and PKR2) that binds PK1 and PK2 with the same affinity. Prokineticin receptors couple to Gas, Gai, and/or Gaq after dimerization [9,10] and induce accumulation of cAMP, activation of extracellular signal-regulated kinases (ERK) and protein kinase B (AKT) and calcium release [11]. PK2β, produced by alternative splicing of pk2 gene, is a biased ligand of prokineticin receptors: indeed, it binds preferentially PKR1 inducing Gas and Gaq and not Gai coupling [12,13].

PKRs are widely distributed throughout the body: PKR1 is mainly expressed in peripheral tissues, including endocrine glands and organs of the reproductive system, spleen, gastrointestinal tract, heart, lungs, and cells of the immune system (such as neutrophils and macrophages). In the CNS, while PKR2 is widely expressed [14], PKR1 is present only in some areas of the brain [15]. PKRs are also expressed on endothelial cells, where they modulate neovascularization (PKR1) and fenestration (PKR2) in adult animals, promoting angiogenesis and vascular permeability [16]. The prokineticin system is involved in a wide range of biological functions: neurogenesis [14], circadian rhythms [17], survival of cardiomyocytes [18], hematopoiesis, and regulation of the immune response [18]. In addition, prokineticins and their receptors are associated with diseases of various tissues: colon, testicular and prostate cancers, polycystic ovary syndrome (PCOS) [19,20,21] and congenital diseases (Kallmann syndrome and Hirschsprung disease) [22,23].

Based on the large amount of data collected over the last twenty years, it is possible to classify prokineticins as chemokines. Like chemokines, prokineticins are small peptides weighing 8–10 KDa that are basic, contain cysteine residues and act as potent chemotactic factors [18]. PK2 released in inflamed peritoneal tissue triggers macrophage recruitment, production of pro-inflammatory cytokines and reduction of anti-inflammatory cytokines through PKR1 activation [24].

PK2 also induces astrocyte migration, which is associated with a gradual shift in astrocytic phenotype A2 [25]. PK2 has been shown to regulate the signaling mechanism between neurons and astrocytes by inducing the phosphorylation of Signal Transducer and Activator of Transcription 3 (STAT3) after subarachnoid hemorrhage, thus acting as an endogenous mechanism for self-repair [26]. Prokineticin receptors are expressed in many tissues and are able to couple different G proteins inducing an extreme variability of responses. For this reason, dysregulation in the prokineticin system can lead to inflammatory and neuroinflammatory conditions, as reported in several studies.

The aim of this review is to describe the prokineticin system in neuroinflammatory conditions such as: inflammatory and neuropathic pain, Alzheimer’s disease, Parkinson’s disease, multiple sclerosis, stroke, obesity, diabetes and gastrointestinal inflammation.

## 2. Inflammatory and Neuropathic Pain

Inflammatory pain is characterized by the release of various inflammatory mediators, such as cytokines and chemokines, from damaged or inflamed tissues and from nociceptive neurons themselves, which induce hypersensitivity both at the site of damage and in adjacent tissues [27].

In the animal model of inflammatory pain induced by the administration of Complete Freund Adjuvant (CFA) in the mouse paw, it has been shown that the development and duration of inflammatory pain correlates temporally with PK2 expression levels in inflamed tissues and that neutrophilic granulocytes are the major source of PK2 [14,28]. Granulocyte colony-stimulating factor (G-CSF), via activation of STAT3, is primarily responsible for PK2 overexpression in neutrophils invading inflamed tissue [29]. PK2 released in inflamed tissue triggers an innate and adaptive immune response. Innate response induces the recruitment of macrophages, the production of pro-inflammatory cytokines (IL-1β and IL-12) and reduction of anti-inflammatory cytokines (IL-10) [14,28]. Adaptative response modulates the cytokine production in T cells which assume a proinflammatory Th1 phenotype [14,28]. Secreted PK2 also regulates angiogenesis and vascular permeability [16,18,19] and induces intracellular Ca^2+^ mobilization, PKCɛ translocation and activation of TRPV1 [14,28], TRPA1 [30], sensitization of P2X channels [31] and suppression of GABAa-activated currents [32].

At the central level, the prokineticin system modulates pain by increasing GABA release in the periaqueductal gray (PAG), thereby increasing the firing activity of On cells and decreasing the firing activity of Off cells in the rostral ventral medulla (RVM) [14,28]. Moreover, PK2 worsens pain perception by suppressing the release of encephalins from neurons in the area postrema (AP), a circumventricular organ rich in PKR2 and lacking BBB [33].

Neuropathic pain is a type of chronic disabling pain due to damage or dysfunction of the peripheral or central nervous system. It is characterized by neuronal oversensitization and abnormal pain perception (allodynia and hyperalgesia). Treatment is currently difficult because the underlying mechanisms are not fully understood, although the involvement of proinflammatory cytokines and chemokines is becoming more apparent.

In animal models of neuropathic pain induced by sciatic nerve injury [spared nerve injury (SNI) and chronic constriction injury (CCI)], damage to the nerve results in tactile allodynia and thermal hyperalgesia, with upregulation of the PK2 and PKR2 receptors in the main stations involved in pain transmission: sciatic nerve, dorsal root ganglia (DRG) and dorsal horns of the spinal cord [14,28]. PK2, which is normally absent in the sciatic nerve, begins to be expressed as early as 3 days after injury in activated Schwann cells and infiltrating macrophages. Subsequently this overexpression spreads towards the DRG and spinal cord at an interval of 10 days after injury. In DRGs, PK2 is detectable in neurons and satellite cells, whereas in the spinal cord it is observed in activated astrocytes [14,28]. PK2 is stored in cytoplasmic vesicles and once released into the synapse by exocytosis, it activates PKR2 receptors at the postsynaptic level [14,28].

**Figure 1:**
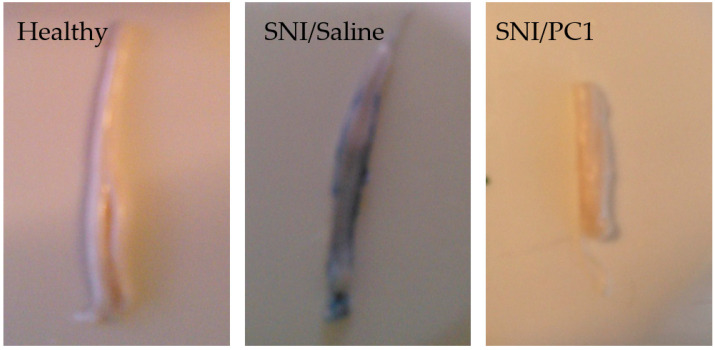
Increased blood–spinal cord barrier permeability (BSCB) after spinal nerve ligation (SNI) in mice. Lumbar spinal cord images of healthy mice compared to injured mice treated with saline or with PC1 (150 µg/kg sc, twice a day). In this Blue Evans assay exudation is evident in the spinal cord of SNI mice but PC1 is able to reduce it already after two days of treatment.

After peripheral nerve injury, PKR2 is also overexpressed in the sciatic nerve, DRG, and spinal cord, whereas PKR1 has overexpression restricted to the nerve [14,28]. The neuropathy induced by peripheral sciatic nerve damage leads to an increase in the permeability of both the “blood–nerve barrier” (BNB) and the “blood–spinal cord barrier” (BSCB) within 24–48 h [34]. The PKRs selective antagonist PC1 is also able to reduce the BSCB permeability; indeed as showed by the Blue Evans assay (Figure 1), the exudation induced by SNI is significantly reduced in the spinal cord after only 2 days of subcutaneous treatment with PC1 [35].

Painful neuropathy is a critical side effect of many chemotherapeutic agents. Mice injected intraperitoneally with bortezomib [36] or vincristine [37] develop allodynia and hyperalgesia, which correlates with strong activation of the prokineticin system activation of macrophage and glial markers, and sustained overproduction of cytokines in the sciatic nerve, DRG, and spinal cord. Moreover, in vitro in DRG neurons, the use of the PKRs antagonist PC1 was shown to prevent the neurotoxic effects of chemotherapeutic agents affecting the reduction in total neurite length induced by bortezomib and vincristine [38]. In vivo, subchronic administration of PC1 in mice exposed to all different models of neuropathy reduces both thermal hyperalgesia and mechanical and tactile allodynia by reducing PK2 overexpression in the different pain stations, restoring physiological levels of pro- and anti-inflammatory cytokines in both the periphery and spinal cord, and reducing spinal glial activation [35,36].

These results indicate that the prokineticin system represents a new potential therapeutic target to combat peripheral neuropathy.

## 3. Neurological Diseases

Several neurological diseases, such as Alzheimer’s disease (AD), Parkinson’s disease (PD) and multiple sclerosis (MS) and stroke have been associated with an exaggerated neuroinflammatory component [1,2]. The PK2 expression levels can be increased by a series of inflammatory and pathological insults such as b-amyloid, reactive oxygen species, hypoxia participating to the progression of the diseases.

### 3.1. Alzheimer’s Disease

Alzheimer’s disease (AD) is characterized by an inflammatory response, both at the level of the brain parenchyma and at the level of the BBB, which follows the deposition of Aβ and increases as the disease progresses [39]. Among the pro-inflammatory signaling molecules released by astrocytes and microglia in AD, a wealth of data suggests that chemokines are produced extensively in AD patients, and these chemokine systems have been shown to be involved in the immune response in the AD brain [40,41]. In addition, alterations in the expression of chemokine receptors on circulating white blood cells from AD patients have been reported, suggesting an influence of the pro-inflammatory milieu in the disease [42].

The prokineticin system, involving the novel chemokine PK2 and its receptors PKR1 and PKR2, is involved in the pathogenesis of AD. It has been found that PK2 can act as a mediator of brain damage and that it plays a crucial role in amyloid-induced neuronal death [43]. Indeed, Aβ_1–42_ increases the mRNA and protein levels of PK2/PKRs in vitro in primary cortical neurons (CNs), and the non-protein PKRs antagonist PC1 protects CNs from Aβ_1–42_-induced neurotoxicity in a dose-dependent manner by reducing PK2 overexpression. Moreover, the use of PC1 prevents the impairment of long-term potentiation (LTP) in the hippocampus of Tg2576 (TG, a transgenic mouse model of AD) mice compared to wild-type (WT) control mice and also reduces AMPA currents [43]. The same group showed in vivo that in a non-transgenic animal model of Alzheimer’s disease, induced by intracerebroventricular (i.c.v.) Aβ_1–42_ administration in rat, the prokineticin system is strongly upregulated in neurons and astrocytes of the hippocampus [43] and that pharmacological blockade of prokineticin receptors with PC1 protects against Aβ_1–42_ induced cognitive deficits by reducing the overexpression of PK2 and PKRs. Subsequent identification of the PKR2 splice variant TM 4–7 in the rat hippocampus led to evidence that this PKR2 isoform is overexpressed in AD and is involved in disease progression [44].

The research was extended to human pathology using human brain tissue samples obtained post mortem from clinically well-documented and neuropathologically confirmed cases of AD. The study showed that expression levels of PK2 in the hippocampus of AD patients are significantly higher than in cognitively normal control subjects. Interestingly, AD patients also have statistically higher serum PK2 levels than healthy individuals, suggesting that PK2 is an important potential biomarker for AD [43].

### 3.2. Parkinson’s Disease

Parkinson’s disease is a common neurodegenerative disorder of the CNS characterized by the loss of dopaminergic neurons in the substantia nigra pars compacta, resulting in a decrease in dopamine concentration in the striatum. Although the exact etiology is not yet clear, neuroinflammation has been identified as a possible trigger of the pathology. Indeed, there are numerous scientific data demonstrating strong microglial activation and cytokine production in patients with PD [45]. A study performed on postmortem brains showed higher levels of cytokines and pro-apoptotic proteins in the striatum and cerebrospinal fluid (CSF) of PD patients than in healthy individuals [46].

A preclinical in vivo study demonstrated that PK2, which is physiologically expressed at very low levels, is significantly upregulated in the striatum of mice with PD. In both the 1-methyl-4-phenyl-1,2,3,6-tetrahydropyridine (MPTP) model and the MitoPark transgenic mouse model of PD, overexpression of PK2 is already observed in the first phase of neuronal degeneration before the onset of motor symptoms. PK2 exhibits neuroprotective functions. By binding to its receptors in dopaminergic neurons, PK2 reduces MPTP-induced neuronal cell death, oxidative stress, and mitochondrial dysfunction. PK2 also promotes mitochondrial biogenesis by upregulating peroxisome proliferator-activated receptor-gamma coactivator (PGC-1) and mitochondrial transcription factor A (TFAM) and activating the survival signaling pathways ERK and AKT. Moreover, intra-striatal administration of PK2 in mice alters the biochemical and behavioral deficits induced by MPTP. Conversely, treatment with the PKRs antagonist PKRA7 enhances the effects induced by MPTP. In the same study, higher levels of PK2 were found in the post-mortem brain and serum of AD patients compared to healthy individuals of the same age [47]. The increase of PK2 in serum correlates with the decrease of lactate in CSF, an index of oxidative stress and mitochondrial dysfunction, indicating an antioxidant function of PK2. At the same time, serum PK2 increase correlates with the increase of Aβ_1–42_ in CSF, indicating a protective role of PK2 at synaptic [48] and also at neuron-astrocyte level [25].

Astrocytes, that become reactive during neurotoxic stress, are evident in the nigro-striatum of PD patients as well as in PD animal models [49]. Astrocytes can transform into a detrimental pro-inflammatory A1 phenotype or a beneficial anti-inflammatory A2 phenotype [50]. PK2 protects dopaminergic neurons in PD, by inducing A2 astrocyte shift and production of neuroprotective factors (such as Nrf2) and increasing glutamate uptake [25].

Taken together, these data on the neuroprotective role of PK2 observed in PD suggest that agonists for PKRs may be new potential therapeutics to counteract the dopaminergic neuronal death that characterizes this pathology. In addition, the tightly regulated serum PK2 level at PD could be used as an early biomarker for the disease.

### 3.3. Multiple Sclerosis

Multiple sclerosis (MS) is an autoimmune and neurodegenerative disease of the CNS characterized by inflammation with demyelination and astroglial proliferation (gliosis) and is the major cause of nontraumatic neurological disability in young adults [51]. The demyelination process is triggered by the inflammatory response involving different cell types such as T lymphocytes, B cells, activated microglia and macrophages, while neurodegeneration is triggered by different processes, such as oxidative stress leading to mitochondrial damage, that are induced or mediated by inflammation [52]. Nowadays, there is a conceptual shift in the understanding of MS immunopathology, away from a purely T- or B-cell mediated role towards the involvement of other cell types. In particular, the role of microglia in repair and remyelination processes has been well described, suggesting that microglia could be a potential target for new drug therapies [53].

PK2 and its receptors are expressed in the CNS [15], bone marrow cells and circulating leukocytes [14]. PK2 induces hematopoietic cell mobilization and differentiation [18] and triggers inflammation [24].

PK2 expression has been studied in mice with experimental autoimmune encephalomyelitis (EAE), an animal model of MS, and in patients with relapsing-remitting disease MS [14]. In EAE mice, PK2 is upregulated in the spinal cord, in inflammatory infiltrated white matter cells, both at mRNA and protein. This PK2 is not only detectable in the CNS, but also in the periphery, lymph node cells (LNC) and serum of EAE mice. Also, in human studies, PK2 levels are significantly higher in the serum of relapsing-remitting MS patients than in healthy individuals, and PK2 transcripts are significantly increased in peripheral blood mononuclear cells (PBMC) [14].

Since macrophages are major components of the CNS inflammatory infiltrates, both in EAE and MS, they could be the source of GCSF-induced PK2 [54]. In both chronic EAE and in relapsing/remitting (Proteolipid Protein, PLP) animal models, preventive and therapeutic treatment with PKRs antagonists, PC1 or PC7, significantly reduces disease severity and CNS inflammation and demyelination. Moreover, treatment with PKR antagonists reduces the production of interferon (IFN)-γ, IL-17 and IL-6, which are deleterious in EAE because they promote the Th17 activation and modulate the autoimmune response against myelin antigen in LNC [14]. In agreement with these data, the same authors showed that PK2 can also enhance Th1 and Th17 responses in vitro, in splenocytes activated against myelin antigen, and that this effect is antagonized by pretreatment with PC7. PK2 promotes a Th1 phenotype by increasing the secretion of IL-1b and IL-12 and reducing the secretion of IL-10 and IL-4 in mouse macrophages and splenocytes [24].

The emerging role of PK2 in MS pathology leads to its identification as an important potential target for future therapeutic application. The goal is to find highly effective and tolerable drugs that are useful especially in the early stages of the disease before permanent disability occurs.

### 3.4. Stroke

The blood–brain barrier is a term used to describe a set of properties possessed by the vasculature of the CNS. The BBB regulates the movement of ions, nutrients, and cells between the blood and the CNS. Disruption of the BBB by occlusion or constriction of blood vessels caused by cerebral ischemia results in decreased nutrient delivery on the one hand and increased movement of water and plasma proteins in the CNS, resulting in the formation of edema, on the other [55,56]. In the ischemic nucleus, ATP depletion due to nutrient deficiency leads to downstream intracellular signaling events such as rapid membrane depolarization resulting in excessive glutamate release, activation of extra-synaptic NMDA receptors, abnormal calcium influx, and formation of reactive oxygen species, ultimately culminating in excitotoxic cell death. In such injuries, hypoxia and the rapid release of glutamate activate a variety of genes that produce proteins with beneficial effects, such as neurotrophins, and others with deleterious effects, such as proinflammatory cytokines. This leads to exacerbation of tissue damage through activation of endothelial cells and macrophages and recruitment of T cells by the BBB [57]. PK2 mRNA expression is induced in the ischemic cortex and striatum following various pathological insults, including hypoxia, reactive oxygen species, and excitotoxic glutamate. After stroke injury, PK2 induction is biphasic: 1–3 h after injury, glutamate induces an initial PK2 overexpression, while a second peak is observed after 24 h, which is due to the activation of hypoxia-inducible factor 1 alpha (HIF1α) [58]. The increase in PK2 levels, which favors inflammation, adversely affects the pathophysiological response to ischemic stroke. Indeed, administration of the PK2 receptor antagonist or silencing of the pk2 gene with RNAi leads to a reduction in infarct volume and blockade of central inflammation. This role of PK2 has recently been confirmed in human studies showing an increase in PK2 expression after ruptures of the abdominal aorta [59]. However, prokineticins may play a dual role in neurodegeneration and neuroprotection. Indeed, PK2 also has a protective effect in apoptotic and necrotic models of cerebral ischemia, in particular it induces the development of ischemic tolerance through the activation of ERK1/2 and AKT signaling [60]. Recently, PK2 was shown to prevent neuronal cell death in traumatic brain injury via ferroptosis by suppressing the synthesis of lipid peroxidation substrates, arachidonic acid phospholipids, the degradation of long-chain fatty acid CoA ligase 4 (Acsl4), and the inhibition of lipid peroxidation [61].

## 4. Control of Energy Metabolism

Alterations of the PK2 pathway determine changes in feeding behavior and in insulin sensitivity influencing the energy homeostasis. Kallmann syndrome (KS) mutations of PK2 and PKR2 genes induce alterations of hormonal reproductive axis, anosmia and may also be related to obesity [22].

### 4.1. Obesity

The hypothalamus controls the stability of body weight through two different neuronal circuits: the POMC/CART and the AgRP/NPY neurons with anorexigenic and orexigenic actions, respectively. The balance between these neuronal circuits oversees the regulation of food intake and energy expenditure and dysregulation leads to the phenomenon of obesity. In a high-fat diet, excess circulating free fatty acids reach the brain at the level of the hypothalamus and trigger local inflammation associated with microglial proliferation and an increase in circulating cytokines. This inflammation in the hypothalamus likely causes synaptic remodeling and neurodegeneration leading to alterations in satiety signals. In this context, pre-adipocytes differentiate and adipocytes increase in size, leading to altered expression of adipokines. This is accompanied by immunological changes as resident macrophages, which have an anti-inflammatory M2-like polarization in lean adipose tissue, transition to an M1-like polarization, leading to the expression of proinflammatory mediators and the recruitment of circulating monocytes [62].

Prokineticin 2, a novel adipokine acting on the G-protein-coupled receptor PKR1, controls food intake [14,63]. The anorexic effects of PK2 depend on the activation of Arcuate Nucleus (ARC) neurons that release melanocyte-stimulating hormone and are modulated by the specific physical interaction of the accessory melanocortin receptor 2 protein (MRAP2) with PKR1 [64,65]. In contrast, peripheral administration of PK2β shows a tendency to increase feeding, likely due to its limited ability to induce STAT3 phosphorylation [66].

PK2 and PKR1 are sensitive to nutritional status: food deprivation for 24 h reduces, while food intake induces their expression in the rat hypothalamus [67]. During the early neonatal period, the expression of PK2 in the hypothalamus is increased in male and female rats, possibly compensating for the immaturity of other appetite-regulating systems. Serum PK2 levels are significantly increased in obese children and in the Chinese population with metabolic syndrome, and this increase correlates with the increase in body mass index (BMI) [68,69]. PK1 is suppressed in metabolically healthy obese patients undergoing bariatric surgery [70]. PK2 also shows anorexic effects after peripheral administration and prevents adipose tissue expansion by suppressing preadipocyte proliferation and differentiation [14,71,72]. Indeed, PKR1-deficient mice show acceleration of preadipocyte proliferation and differentiation. Adipose tissue expansion creates a hypoxic environment that links and reciprocally regulates angiogenesis and inflammation through the activation of HIF1α, regulating the expression of VEGF as well as of prokineticins [71,73].

PK2 has been shown to promote the inflammatory phenotype of mouse macrophages and to reduce the production of anti-inflammatory cytokines IL-10 and IL-4 in mouse splenocytes [24]. It is likely that PK2 could play a role in triggering the phenotypic switch from M1 to M2 and maintaining M2 polarization to maintain adequate adipocyte function, but the exact role of PK2 in this process remains to be elucidated. PK2 activating PKR1 signaling reduces food intake [74], suggesting that selective PKR1 agonists may have therapeutic potential for the treatment of obesity.

### 4.2. Diabetes

Type 2 diabetes (T2D) is caused by loss of insulin sensitivity in adipocytes, muscle, and other insulin-dependent cells, leading to loss of effective control of glucose concentration, resulting in inflammation and disruption of metabolic pathways. A monolayer of endothelial cells separates the interstitium from blood and lymph and determines the bidirectional transfer of solutes, macromolecules, and insulin across the blood barrier (BB) to metabolically active tissues such as muscle, fat, and brain. In diabetes, chronic low-grade inflammation promoted by proinflammatory cytokines and oxidative stress impairs the integrity of the BB and alters the diffusion of insulin and nutrients from the blood to tissues, leading to important pathological consequences of diabetes such as cardiac damage, infertility, and pregnancy complications [75].

The insulin receptor IRS1 node activates two important signaling pathways, ERK and AKT, which control cell growth and metabolism by regulating glucose uptake and utilization. In diabetes, the increase in inflammatory cytokines leads to dysregulation of IRS1 phosphorylation, which impairs insulin signal transduction in favor of ERK signaling pathways while blocking AKT activation.

In humans, plasma PK2 levels are negatively correlated with T2D patients and a common Single Nucleotide Polymorphism (SNP) of the pk2 gene has been associated with T2D [76]. PKR1 plays an essential role in regulating endothelial insulin uptake and capillary formation. The mouse model with endothelial-specific PKR1 loss-of-function shows decreased insulin uptake in endothelial cells and decreased AKT activation, as well as defective expression and activation of oxidative nitrogen synthase [77]. PKR1 also regulates the release of insulin via endothelial cells into skeletal muscle, a limiting step in glucose uptake. In skeletal muscle, PKR1 is downregulated under conditions of insulin resistance and AKT phosphorylation is reduced [78].

Metformin (Met) is a widely used treatment for T2D because it induces glucose uptake by activating AMP kinase. Met administration induces the expression of PK2, PKR1, and PKR2 in cardiomyocytes and testis, which provides beneficial effects against diabetes-related cardiac and testicular damage by regulating the AKT/GSK3β pathway. This finding confirms previous data showing that PK2 is involved in cardiac muscle survival and angiogenesis through the AKT and STAT3 pathway [79,80]. Insulin resistance leads to secondary hyperinsulinemia, which is crucial for pregnancy complications such as recurrent miscarriage and preeclampsia, as well as metabolic disorders such as polycystic ovary syndrome (PCOS). Insulin, via HIF1α, regulates the expression of the pro-angiogenic factor PK1 in stromal cells of the human endometrium, affecting their migration and consequently the proper implantation of the embryo. Dihydrotestosterone (DHT), but not testosterone, enhances the effect of insulin in increasing PK1 levels [81]. Diabetes leads to severe neuroinflammatory complications such as neuropathic pain. In the animal model of diabetic neuropathy induced by streptozotocin in mice, an increase in the expression of PK2 and its receptors PKR1 and PKR2 has been demonstrated in the sciatic nerve and spinal cord, and this overexpression correlates with the development of mechanical allodynia [82]. Treatment with the PC1 antagonist normalizes prokineticin levels, restores the balance of pro-anti-inflammatory cytokines, and reduces peripheral immune activation in the spinal cord and nerves of diabetic mice, thereby blocking both the allodynia and the inflammatory events underlying the disease [82].

## 5. Gastro-Intestinal Inflammation

The enteric nervous system (ENS), a major division of the peripheral nervous system, consists of an extensive network of enteric and glial neurons that regulate various processes in the gastrointestinal tract, including motility, local blood flow, transport, and mucosal secretion. It comprehends microcircuits of primary afferent neurons that respond to local stimuli and integrate information coordinating motor output. The ENS therefore has unique sensory and motor properties.

ENS is derived from stem cells from the neural crest that migrate into and along the primary intestine. Defects in the establishment of the ENS cause enteric neuropathies, including Hirschsprung’s disease (HSCR), which is characterized by the absence of enteric neural crest cells (NCC) in the distal part of the colon. Prokineticin signal transduction system is present in human enteric neural crest cells and, interacting with Glial Cell line-Derived Neurotrophic Factor (GDNF) and RET signaling, provides an additional level of signaling to maintain enteric NCC proliferation and differentiation [83]. RET (rearranged during transfection) is a transmembrane glycoprotein receptor-tyrosine kinase involved in the development of the kidneys and enteric nervous system during embryogenesis. RET binding the complex of the GDNF family ligands with GDNF family receptors activates several signal transduction cascades involved in cellular proliferation, including the MAPK and STAT3 pathways [84].

Mutational analysis, in a cohort of HSCR patients, evidenced sequence variants of pkr1, pkr2, pk1 and pk2 genes, that in some cases are present in combination with GDNF or RET mutations, providing the first evidence to consider them as susceptibility genes for HSCR [23].

As mentioned previously, prokineticins have been named for their ability to powerfully contract longitudinal smooth muscle strips of ileal guinea pigs [5]. In addition, oral PK1 administration increases the secretion of intestinal fluid by activating the prostaglandin receptors [85]. These observations, together with the discovery that PK2 can modulate intestinal ion transport, raises the possibility that PK2 inhibitors may have some clinical utility in gastrointestinal disorders, such as irritable bowel syndrome and inflammatory bowel disease [86].

Pk2 gene expression is strongly upregulated in biopsy specimens from patients with ulcerative colitis (RCU) and similar increases have been observed in animal models of inflammatory colitis [87,88]. Maternal exposure to low levels of corticosterone during lactations, makes the offspring (CORT-nursed rats) protected against 2,4,6-Trinitrobenzenesulfonic acid (TNBS)-induced colitis [88]. This reduction could be ascribed to less activation of NF-κB involved in the expression of Pk2 gene [88,89].

Elevated PK2 levels, as a consequence of inflammation of the gastrointestinal tract, also induce visceral pain through prokineticin receptors; indeed, a PKR antagonist is able to reverse inflammatory visceral pain [86].

## 6. Conclusions

Neuroinflammation is defined as an inflammatory response within the brain or the spinal cord associated with activation of the glia with significant production of cytokines and chemokines, infiltration of peripheral immune cells, edema, increased permeability and disruption of the blood–brain barrier. This damage can lead to infiltration of immune cells into the brain with consequent exacerbation of central inflammation.

Neuroinflammation, which is seen in obesity, diabetes, Parkinson’s and Alzheimer’s, causes several overlapping neurodegenerative mechanisms, including oxidative stress, mitochondrial dysfunction, and inflammation [90,91].

**Figure 2:**
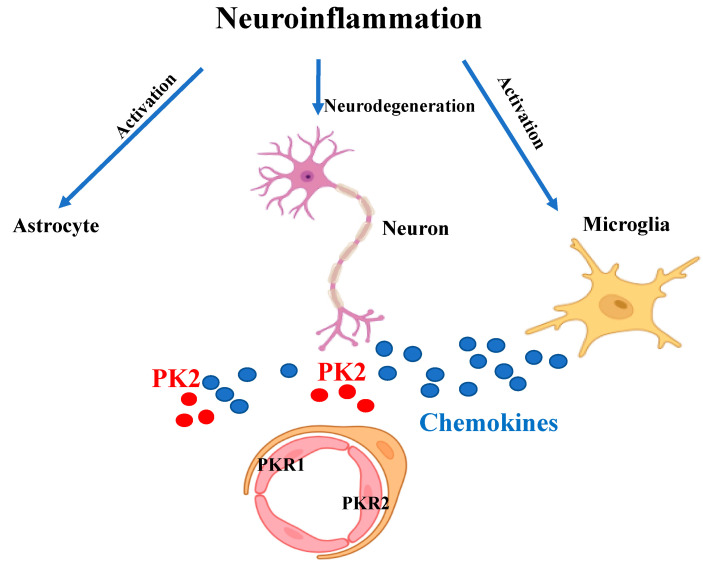
Schematic representation of the inflammatory state. Neuroinflammation leads to neurodegeneration and to over-activation of microglia and astrocytes which release pro-inflammatory cytokines and chemokines (blue circles). The chemokine-like protein PK2 (red circles) is secreted by neurons and astrocytes. Upon binding to PKR1 and PKR2 receptors on endothelial cells it promotes angiogenesis and vascular permeability.

The prokineticin system is a central players in neuroinflammation (Figure 2) and could be a novel therapeutic target for neuroinflammatory diseases. However, since the system is present in different tissues and is involved in numerous physiological processes, the use of a drug capable of antagonizing the pro-inflammatory effect of prokineticins could also induce the onset of side effects.

## Data Availability

Not applicable.
